# Novel tools for the surveillance and control of dengue: findings by the DengueTools research consortium

**DOI:** 10.1080/16549716.2018.1549930

**Published:** 2018-12-03

**Authors:** Annelies Wilder-Smith, Hasitha Tissera, Sazaly AbuBakar, Pattamaporn Kittayapong, James Logan, Andreas Neumayr, Joacim Rocklöv, Peter Byass, Valérie R. Louis, Yesim Tozan, Eduardo Massad, Raman Preet

**Affiliations:** a Unit of Epidemiology and Global Health, Department of Public Health and Clinical Medicine, Umeå University, Umeå, Sweden; b Epidemiological Unit, Ministry of Health, Colombo, Sri Lanka; c WHO Collaborating Centre for Arbovirus Reference and Research (Dengue/Severe Dengue), Tropical Infectious Diseases Research and Education Centre (TIDREC) University of Malaya, Kuala Lumpur, Malaysia; d Center of Excellence for Vectors and Vector-Borne Diseases, Department of Biology, Faculty of Science, Mahidol University, Salaya, Nakhon Pathom, Bangkok, Thailand; e Department of Disease Control, London School of Hygiene and Tropical Medicine, London, UK; f Department of Medical Services, Swiss Tropical and Public Health Institute, Basel, Switzerland; g Heidelberg Institute of Global Health, Heidelberg University Medical School, Heidelberg, Germany; h NYU College of Global Public Health, New York, NY, USA; i School of Applied Mathematics, Fundacao Getulio Vargas, Rio de Janeiro, Brazil

**Keywords:** Dengue, DengueTools, surveillance, impregnated clothing, schools, Aedes, vectorial capacity, predictive modelling, importation, travel, Zika, reverse transcription-recombinase polymerase amplification

## Abstract

**Background**: Dengue fever persists as a major global disease burden, and may increase as a consequence of climate change. Along with other measures, research actions to improve diagnosis, surveillance, prevention, and predictive models are highly relevant. The European Commission funded the DengueTools consortium to lead a major initiative in these areas, and this review synthesises the outputs and findings of this work conducted from 2011 to 2016.

**Research areas**: DengueTools organised its work into three research areas, namely [1] Early warning and surveillance systems; [2] Strategies to prevent dengue in children; and [3] Predictive models for the global spread of dengue.

Research area 1 focused on case-studies undertaken in Sri Lanka, including developing laboratory-based sentinel surveillance, evaluating economic impact, identifying drivers of transmission intensity, evaluating outbreak prediction capacity and developing diagnostic capacity. Research area 2 addressed preventing dengue transmission in school children, with case-studies undertaken in Thailand. Insecticide-treated school uniforms represented an intriguing potential approach, with some encouraging results, but which were overshadowed by a lack of persistence of insecticide on the uniforms with repeated washing. Research area 3 evaluated potential global spread of dengue, particularly into dengue-naïve areas such as Europe. The role of international travel, changing boundaries of vectors, developing models of vectorial capacity under different climate change scenarios and strategies for vector control in outbreaks was all evaluated.

**Concluding remarks**: DengueTools was able to make significant advances in methods for understanding and controlling dengue transmission in a range of settings. These will have implications for public health agendas to counteract dengue, including vaccination programmes.

**Outlook**: Towards the end of the DengueTools project, Zika virus emerged as an unexpected epidemic in the central and southern America. Given the similarities between the dengue and Zika viruses, with vectors in common, some of the DengueTools thinking translated readily into the Zika situation.

## Background

Despite the increasing global burden of dengue both in terms of annual incidence and geographic distribution [1,2], dengue remains a neglected disease [3]. In 2010, the European Commission launched a Seventh Framework Programme call entitled ‘Comprehensive control of dengue under changing climatic conditions’. In response, scientists from 14 institutions in Europe, Asia, and South America formed the “DengueTools” consortium. DengueTools was awarded a grant in 2011, together with two other dengue research consortia (IDAMS and DENFREE) [4]. DengueTools’ objectives were to achieve better diagnosis, surveillance, prevention, and predictive models and improve our understanding of the spread of dengue to previously uninfected regions (including Europe) in the context of globalisation and climate change. The consortium comprised 12 work packages to address research objectives in three main areas:

- Research area 1: Early warning and surveillance systems.
- Research area 2: Strategies to prevent dengue in children.
- Research area 3: Predictive models for the global spread of dengue, in particular the risk of introduction and establishment in Europe.

The composition of the consortium including its institutional geography, work packages and research design are outlined in detail in the DengueTools study design paper from 2012 [5]. Here, in compliance with accountability and transparency on best use of European Commission research funding, we synthesise the main research findings and publications from the five-year DengueTools research project.

## Research area 1

***Overall objective: Develop a comprehensive, early warning, surveillance system that has predictive capability for epidemic dengue and benefits from novel tools for laboratory diagnosis and vector monitoring***

Rationale: The dramatic global spread and increased frequency and magnitude of epidemic dengue in the past 50 years underscores the critical need for more effective surveillance, including better tools.

## Specific objectives

### Develop a laboratory-based sentinel disease surveillance system in Sri Lanka

Dengue poses a significant disease burden in Sri Lanka [6]. Historically surveillance was passive, with mandatory dengue notifications based on clinical diagnosis and limited laboratory confirmation. With DengueTools funds, we established laboratory-based enhanced sentinel surveillance in Colombo District [7]. Three outpatient clinics and three government hospitals from the Colombo metropolitan area were selected. We enrolled 3,127 febrile cases, and found that 44% were PCR and/or NS1 positive for dengue (of which 54% were inpatients and 7.6% in outpatients). Dengue serotype 1 (DENV-1) was responsible for 85% of the cases and serotype 4 (DENV-4) for 15%. The incidence of severe dengue in those hospitalised was 22%, with a case fatality rate of 0.9% in those presenting with severe dengue. The case fatality rate in the cohort was below 0.1%.

The emergence of DENV-1 as the foremost serotype highlights the changing epidemiology of dengue and the need for continued surveillance and prevention [8]. DENV-4 infections are globally less common, and also infrequent in Sri Lanka. As about 15% of our population had a DENV-4 infection, to better understand the molecular epidemiology of DENV-4 infections in Sri Lanka, we conducted whole-genome sequencing on dengue patient samples from two different geographic locations, showing the DENV-4 strains belonged to genotype 1, most closely related to those circulating in India and Pakistan [9].

We also explored internet-based media coverage in Sri Lanka to assess whether internet hits would correlate with dengue activity. Digital media coverage for dengue was far higher than for other infectious diseases and highly correlated with national epidemiological trends in dengue [10]. Our findings underpin previous claims that digital media reports of dengue reflect national epidemiological trends, both annually and in inter-annual seasonal variations, thus acting as proxy bio-surveillance to provide early warning and situation awareness.

### Evaluate the economic impact of dengue

In Sri Lanka, the continuous involvement of large numbers of public health personnel in dengue control and the provision of free medical care to dengue patients place a formidable financial burden on the Ministry of Health. We retrospectively estimated the public sector costs of dengue control and hospitalisations in Colombo district. In the epidemic year of 2012, the total cost was estimated at US$3.45 million (US$1.50 per capita), of which US$.42 was for control. Personnel costs accounted 79% of dengue control and 46% of hospitalisations [11]. Average costs per hospitalisation ranged between US$216–609 and US$196–866 per paediatric and adult patient respectively, by disease severity and treatment setting. Country-specific evidence on the economic burden of dengue is important to set public health priorities and decide about deploying existing or new technologies.

### Investigate socio-behavioural, entomological and environmental indicators that drive dengue transmission intensity in Sri Lanka

To assess *Aedes* mosquito breeding sites and prevention practices of community members, we conducted a cross-sectional entomological survey in 1,469 premises (1,341 households, 11 schools, 99 work or public sites, 5 open lands, and 13 non-specified) located in a sub-district of Colombo [12]. Types of breeding sites and infestation with larvae or pupae were recorded. A questionnaire on dengue vector control practices was administered to occupants. Schools had a higher proportion of productive sites (64%) than households (15%). The odds ratio (OR) for productive breeding sites was 2.77 (95% CI 1.58–4.86) for schools and other non-residential buildings compared to residences. Residential occupants were more likely to take preventive measures. These findings emphasise the need to expand public health strategies to schools and other non-residential premises.

### Evaluate the outbreak prediction capability of weather in Sri Lanka

Understanding the drivers of dengue is vital in controlling and preventing the disease spread. Weekly weather and dengue data, measured in 10 divisions in Kalutara, a district in Sri Lanka were retrieved and analysed [13]. Distributed lag non-linear time series analysis was used to estimate division-specific risks, and hierarchical models were fitted to estimate the overall relationships between el Niño conditions, weather and dengue across divisions. A clear relationship was observed between Oceanic Nino Index (ONI) and dengue in Kalutara district, Sri Lanka, with a peak effect at 6 months. The pattern between ONI and dengue incidence was mediated by rainfall and temperature. Consistent exposure-response patterns between different geographical locations were observed for rainfall, showing increasing relative risks of dengue with increasing rainfall from 50 mm per week, with the highest risk 6 to 10 weeks after rainfalls of more than 300 mm per week. The overall relative risk of dengue associated with temperature increased steadily from lower temperatures towards the mean value of 29.8°C, starting from a lag of 4 weeks, and increased for temperatures above the mean value with a cumulative growing association with lags up to 12 weeks.

### Develop and validate novel diagnostic assays for point-of-care use

Diagnostics for dengue is challenging without affordable, sensitive and specific point-of-care testing [14]. We developed a prototype reverse transcription-recombinase polymerase amplification (RT-RPA) assay that is quick and can be undertaken in low resource settings [15]. The RPA is an isothermal nucleic acid amplification technology where reactions take place at a low and constant temperature between 22°C and 45°C [16]. Fluorescence was detected using a portable real-time fluorometer, ‘Twista®’, which has a small footprint and can be rendered entirely portable with the addition of a rechargeable battery pack, developed by the private sector collaborator, TwistDx.

The assay detected as few as 10 copies of DENV RNA within 20 minutes without the need for thermocycling amplification. The RT-RPA assay is the most rapid molecular diagnostic tool available for the detection of DENV. The RT-RPA assay showed good concordance (κ = 0.723) with the RT-LAMP and qRT-PCR assays in detecting DENV in viraemic samples [17]. When evaluated for effectiveness and efficiency among new users, the user satisfaction towards the RT-RPA protocol and interpretation of results were 100% and 90%, respectively [18]. Though the RPA requires use of the TwistDx equipment, the RPA is simple, affordable, user-friendly, and independent of outside temperature, rendering it suitable for field-use.

### *Develop novel assays for virus detection and characterisation in* aedes *mosquitoes*

Viral detection in *Aedes* mosquitoes is often difficult, especially for small amounts of DENV in batches of mosquitoes from dengue surveillance. We developed a new polymerase chain displacement reaction (PCDR), which uses multiple nested primers in a rapid, capped, one-tube reaction that increases the sensitivity of normal quantitative PCR (qPCR) assays [19]. Sensitivity increased approximately 10-fold. Increased sensitivity will be useful in nucleic acid detection for viral diagnostics, and can enhance virus detection in vector surveillance.

## Research area 2

***Overall objective: Develop novel tools for the prevention of dengue in school-aged children***

Rationale: Compliance with personal protective measures such as insect repellents [20] are low [21]. *Wolbachia*-infected mosquitoes or genetically modified male mosquitoes appear promising [22,23], but their use remains controversial and large-scale implementations are unlikely in the near future. Hence, there is an urgent need for timely scalable solutions. Since *Aedes aegypti* is a day-biting mosquito, developing technologies that offer daytime protection against mosquito bites is essential. Permethrin is a pyrethroid-based insecticide registered by the US Environmental Protection Agency since 1977 that has been extensively used as an insect repellent and insecticide with a documented safety record [24]. Permethrin can be bound to fabric fibres in clothing via different techniques such as micro-encapsulation and polymer coating [25,26].

## Specific objectives

### Review literature on protective efficacy of insecticide-treated clothing against vector-borne diseases

Insecticide-treated clothing has been used for many years by the military and in recreational activities as personal protection against bites from a variety of arthropods including ticks, chigger mites, sandflies and mosquitoes and is thought to be safe [24]. We reviewed the evidence base for its use against bites from arthropods and its effect on arthropod-borne pathogen transmission. Insecticide-treated clothing has been reported to provide between 0% and 75% protection against malaria and between 0% and 79% protection against leishmaniasis [24]. No field trials had been conducted to demonstrate the efficacy against *Aedes*-transmitted diseases. However, one study showed that wearing permethrin-treated clothing resulted in a reduction in the number of *Aedes* bites by 90% [27] suggesting that it could potentially be a promising intervention for *Aedes*-transmitted diseases such as dengue.

### Determine the efficacy of insecticide-treated school uniforms (isus) under different laboratory scenarios

Here we assessed the efficacy and durability of different types of ISUs on laboratory-reared *Aedes aegypti* [28]. Standardised World Health Organization Pesticide Evaluation Scheme (WHOPES) cone tests were used to assess knockdown (KD) and mortality of *Aedes aegypti* tested against factory-treated fabric, home-dipped fabric and microencapsulated fabric. The standardised **cone test** is used to assess the effectiveness of an insecticide and its persistence on materials, following the WHO protocol. This **test** aims to compare the behaviour of mosquitoes while in contact with treated mosquito nets. We found InsectShield®-treated fabric achieved the highest KD and mosquito mortality, and decided to use InsectShield® for the school-based randomised controlled trial.

In arm-in-cage experiments whereby volunteers hold their arm in cages for starved laboratory bred Aedes mosquitoes to feed on showed that ISUs reduced landing by 58.9% and biting by 28.5% [29]. We assessed the effect of washing on treated clothing, skin coverage and protection against resistant and susceptible *Aedes aegypti* using modified WHO arm-in-cage assays. Coverage was further assessed using free-flight room tests to investigate the protective efficacy of unwashed factory-treated clothing. Clothing was worn as full coverage (long sleeves and trousers) and partial coverage (short sleeves and shorts). In the arm-in-cage assays, unwashed clothing reduced landing by 58.9% and biting by 28.5% [29]. Landing and biting for resistant and susceptible strains was not significantly different. In free-flight room tests, full coverage treated clothing reduced landing by 24.3% and biting by 91% with partial coverage reducing landing and biting by 26.4% and 49.3% respectively, thus coverage type made no significant difference on landing [29]. Whilst ISUs with long sleeves and trousers provided the highest form of protection, partially covering the body with permethrin-treated clothing did provide significant protection against biting.

### Model the potential impact of isus on reducing dengue incidence

Mathematical models [30] were used to calculate the risk of dengue infection based on force of infection, taking into account the estimated proportion of mosquito bites that occur in school and the proportion of school time that children wear ISUs, plus the findings from our laboratory studies on the impact of reduction on mosquito bites [29]. We modelled the use of ISUs as having an efficacy varying from around 6% in the most pessimistic estimations, to 55% in the most optimistic scenarios simulated. The efficacy of ISUs would depend on the compliance of the target population in terms of proper and consistent wearing of ISUs.

### Conduct a school-based randomised controlled trial to assess the efficacy of isus on the reduction of dengue incidence in school-aged children in Thailand

As children spend a considerable amount of their day at schools, strategies that reduce human–mosquito contact to protect against day-biting *Aedes* mosquitoes could be effective. Most schools in dengue endemic countries require school uniforms. We hypothesised that ISUs might reduce dengue infections [31]. We tested our hypothesis by setting up a large community-based trial to determine the reduction of dengue infections due to ISUs (InsectShield®) [32].

We ran a blinded trial over a school term (5 months) in 10 schools in Thailand (5 intervention and 5 control) [33]. The primary endpoint was laboratory-confirmed dengue infections. We also included as secondary endpoints 1-hour knock-down and 24 hour mosquito mortality on ISUs measured by standardised WHOPES bioassay cone tests at baseline and after repeated washing. Entomological assessments were done inside classrooms and in school environments.

Paired serum samples were obtained from 1,655 pupils [33]. We did not find a significant difference in proportions of students having incident dengue infections between the intervention and control schools, allowing for clustering by school. WHOPES cone tests showed a 100% knock down and mortality of *Aedes aegypti* mosquitoes exposed to impregnated clothing at baseline and up to 4 washes, but this efficacy rapidly declined to below 20% after 20 washes. Results of the entomological assessments showed that the mean number of *Aedes aegypti* mosquitoes caught inside the classrooms of the intervention schools was significantly reduced in the month following the introduction of the impregnated uniforms, compared to those collected in classrooms of the control schools, but there were no differences in the subsequent months.

## Assess the acceptability of ISUs

Using a mixed method approach with qualitative and quantitative approaches to survey Thai parents, we found that the acceptability of ISUs was high. Most parents (87.3%; 95% CI 82.9–90.8) would allow children to wear ISUs and 59.9% (95% CI 53.7–65.9) of parents would accept additional costs for ISUs over normal uniforms, should ISUs be shown to be effective against mosquito bites and reduction of dengue [34].

### Develop a cost-effectiveness framework and model for school-based preventive interventions using isus

We investigated the cost-effectiveness of using ISUs for population-based dengue prevention in school-aged children under various scenarios of intervention effectiveness and cost and dengue infection risk, using data specific to Thailand. At an average incidence rate of 5.8% per year, the intervention proved cost-effective (cost per disability-adjusted life-year averted ≤$16,440) when the intervention cost per child per year was $5.30 or less and the intervention effectiveness was 50% or higher, and cost saving in all scenarios in which the cost was $2.90 or less and the effectiveness was 50% or higher [35]. The intervention would be of no interest to Thai policy makers if the per-child cost were $10.60 or higher per year regardless of its effectiveness.

## Research area 3

***Overall objective: Explore reasons for the spread of dengue, in particular the risk of introduction and establishment in Europe, within the context of vectorial capacity, human mobility, and climate change, using predictive models.***

Rationale: Long-term climate change and weather variability affect the proliferation of *Aedes* mosquitoes. Furthermore, air travel between dengue-endemic countries and from dengue-endemic to non-endemic vulnerable settings has increased exponentially [36]. Whilst imported dengue cases to the US have resulted in small dengue clusters for many years [37], the first sporadic autochthonous cases in Europe (France and Croatia) were reported in 2010 [38,39]. In 2012, the first major European outbreak of dengue occurred in Madeira [40]. International travellers are increasingly at risk of dengue [2,41–44], with attack rates reported as high as 5.51 cases per 1,000 travel-months to endemic areas [45]. Dengue is now the leading cause of fever in returning travellers, having overtaken malaria for travellers to South East Asia [46].

## Specific objectives

### Describe dengue importations into dengue-naïve countries (with a focus on Europe)

Between 6–87% of travellers become ill during travel [47]. Sentinel surveillance can aid to estimate the burden of disease in travellers [48–54] despite the lack of a denominator [55]. We conducted sentinel surveillance in travellers returning to Europe through travel medicine providers from TropNet. TropNet is a European Network on Imported Infectious Disease Surveillance that conducts sentinel surveillance in ill-returned travellers seen by travel medicine providers of the network. Our sentinel surveillance system included 242 returning dengue viraemic travellers; corresponding to 36% of all returning travellers with dengue. Consequently, a significant number of viraemic dengue-infected travellers return to Europe, underpinning the potential for the introduction of dengue viruses into dengue-naïve areas [56]. The most likely place for acquiring dengue was Asia, followed by the Americas, then Western Pacific region, and the smallest number from Africa [57].

First, we detected circulation of DENV-3 (genotype III) in Cuba in 2013 and 2014, despite the fact that PAHO did not report any dengue during that time period. The last detection of that genotype in Cuba was during a big epidemic occurring in 2001–2002 (unpublished data). Second, we isolated a DENV-3 strain from a traveller returning from Togo and Burkina Faso, both countries where circulation of dengue had not been reported, highlighting that travellers can unmask ongoing transmission. We also identified a dengue virus strain from Angola in 2013 unmasking a new outbreak in that country [58]. Third, virus isolates obtained from travellers who had acquired dengue in Madeira, Portugal, were sequenced and found to be genetically linked to virus isolates from Brazil and Venezuela [40,59]. We developed an importation index that takes into account both travel volume and the extent of dengue incidence in Brazil and Venezuela which pointed towards Venezuela being the source of importation [60]. Fourth, DENV-1 (genotype I, Asian) was isolated from a traveller returning from Japan during Tokyo’s first dengue outbreak of about160 autochthonous cases in August-September 2014. Investigating the origin of the Japanese outbreak, sequencing suggested that this virus may have been imported from China, Indonesia, Singapore, or Vietnam [61]. Based on importation modelling, China was identified to be the most likely source [61].

### Model dengue virus introductions via travellers

To estimate the risk of travellers introducing dengue, travel volume, incidence of dengue in the country of disembarkation, probability of being infected at the time of travel and duration of viraemia need to be taken into account. We developed mathematical models for the risk of introducing dengue into dengue naïve areas [62]. These models were applied to various situations: risk of importation of dengue into Italy [63] given the presence of susceptible *Aedes* populations that allowed an outbreak of chikungunya to occur in 2007 [64]; the risk from non-immune persons travelling to Thailand [65]; and to other infectious diseases such as polio [66,67] and Zika [68,69].

The mathematical modelling was also applied to estimate the number of dengue-viraemic air passengers from the main dengue-endemic countries entering Europe. Our models estimate a range of zero to 167 dengue-viraemic air passengers entering the 27 receiving countries in one year, with Germany receiving the highest number followed by France and the UK [70]. Modelling the number of importations, the presence of suitable mosquito populations and vectorial capacity, the risk of onward transmission in Europe is reassuringly low [70].

### *Model vectorial capacity for* aedes *mosquitoes in temperate climate zones and globally under current and future climate scenarios*

Vectorial capacity describes a vector’s propensity to transmit dengue taking into account human, virus, and vector interactions. Recent evidence shows that diurnal temperature range (DTR) plays an important role in influencing *Aedes* vector competence. In these studies, we developed predictive models of vectorial capacity for *Ae. aegypti* and *Ae. albopictus* [71,72], ability to transmit dengue in relation to temperature and diurnal temperature variability. We found strong temperature dependent dengue epidemic potential, with optimal transmission around a constant temperature of 29⁰C, with vectorial capacity decreasing at higher temperatures. This decline was partly offset when diurnal temperature range was larger, with at least part of the day reaching near-optimal conditions [71,72]. Incorporating these findings using historical and predicted temperature and DTR over 200 years (1901–2099), there was an increasing trend in dengue epidemic potential in temperate regions. Future changes depend considerably on the current and future carbon emissions. Southern Europe is at risk of increasing seasonal dengue transmission windows in future climate change scenarios.

### *Assess efficacy of ULV and thermal aerosols of deltamethrin for control of* aedes albopictus *in Europe*

Ultra-low volume (ULV) insecticidal aerosols dispensed from vehicle-mounted cold-foggers are widely considered the method of choice for control of *Ae. aegypti* and *Ae. albopictus* Nevertheless, their effectiveness has been poorly studied, particularly in Europe. We evaluated the efficiency of the two widely used space spraying methods to control *Ae. albopictus* and *Ae. aegypti* in France, and found hand-held thermal foggers to be more effective than ULV [73].

### Develop geo-spatial modelling and risk maps

A systematic literature review was undertaken to study predictors and modelling approaches used to create dengue risk maps [74]. The relevant predictors among demographic and socio-economic variables were age, gender, education, housing conditions and level of income. Among environmental variables, precipitation and air temperature were often significant predictors. Descriptive maps showing dengue case hotspots were useful for identifying high-risk areas. Predictive maps based on more complex methodology facilitated advanced data analysis and visualisation. No specific patterns were identified in the combination of predictors or models across studies.

### Explore the contribution of urbanisation to dengue transmission

Globalisation with increasing interconnectivity, ecological habitat encroachment, socioeconomic changes, climate change, virus evolution, and urbanisation all drive dengue transmission intensity [75]. We studied the relative contributions of putative drivers for the rise of dengue in Singapore: population growth, climate parameters and international air passenger arrivals from dengue endemic countries, from 1974 to 2011 [76]. Estimating the extent of the contribution of three factors (population growth versus weather variability versus air passenger arrivals into Singapore) on the increasing dengue incidence, we found that population growth contributed to 86% while the residual 14% was explained by increase in temperature. No correlation was found with incoming air passenger arrivals into Singapore from dengue endemic countries.

## Concluding remarks

As a result of the EU funded laboratory-enhanced surveillance project for laboratory capacity building, a dedicated government laboratory in Sri Lanka was set up to perform routine molecular testing for dengue which operated sustainably after the funding period. Quality assurance with a subset of samples sent to the Duke-NUS Laboratory ‘Emerging Infectious Diseases Program’ showed high agreement [7]. The improved RT-RPA as a rapid assay for dengue diagnosis is now routinely used for detection of DENV at the WHO Collaborating Center for Arbovirus Reference and Research (Dengue/Severe Dengue) at the University of Malay.

Given the day-biting behaviour of *Aedes* mosquitoes, ISUs could potentially be a novel tool to reduce mosquito-borne diseases and local vector populations. Our results from the WHOPES cone tests underpin the potential for ISUs to protect against dengue by reducing the populations of *Aedes* mosquitoes and hence mosquito-bites: knock-down effect and mortality immediately after impregnation of uniforms with permethrin by the InsectShield® proprietary method were close to 100% [33], consistent with results obtained under laboratory conditions [28]. Furthermore, we documented a significant reduction in *Aedes* mosquito numbers in the classrooms of the intervention schools in the first month after the start of the trial at the time when the insecticidal activity of impregnated uniforms was still 100%.

However, our cluster-randomised controlled trial in ten Thai schools involving 1,811 children did not show serological evidence of a protective effect over the 5-month study period. This negative result was probably due to the rapidly waning efficacy of InsectShield® ISUs under field conditions. After 20 washes, the knock-down and mortality effects on mosquitoes were well below 20%. Long-lasting insecticide-treated bed-nets were a major breakthrough in the control of malaria. However, bed-nets do not get washed so frequently. For ISUs to be a viable public health intervention they must withstand regular washing. If the rapid washing-out of permethrin can be overcome by novel technological approaches, ISUs would deserve to be re-evaluated as a potentially cost-effective and scalable intervention [33].

Both the questionnaire survey in Sri Lanka [12] and clustering analyses in Thailand [77] found that schools are high-risk locations for dengue transmission, underpinning the urgent need to implement vector control strategies at schools, while maintaining household efforts.

Many unknowns, including effective entomological predictors, genetic diversity of circulating viruses, population serological profile, and human mobility still limit the ability to produce accurate and effective risk maps, and impede the development of early warning systems [74]. However, our newly developed mathematical models and vectorial capacity calculations serve as additional tools to assist to predict the risk of importation and potential for establishment in dengue naïve areas.

Under various climate change scenarios, seasonal peaks and time windows for dengue epidemic potential will increase during the 21st century in Europe. We mapped dengue epidemic potential in Europe and identified seasonal time windows when major cities are most at risk of dengue transmission from 1901 to 2099 [71]. Our findings illustrate, that besides vector control, mitigating greenhouse gas emissions crucially reduces the future epidemic potential of dengue in Europe. Much still needs to be learned about the impact of climate on dengue transmission.

Models also showed that population growth is the main driver for dengue resurgence in Singapore [76]. These findings have significant implications for predicting future trends of the dengue epidemics given the rapid urbanisation with population growth in many dengue endemic countries. It is time for policy-makers and the scientific community alike to pay more attention to the negative impact of urbanisation and urban climate on diseases such as dengue.

Increasing dengue burdens and risks justify dengue vaccination. Our literature review showed that country-level analytical expertise in economic analyses needs to be strengthened to facilitate evidence-based decision-making on dengue vaccine introduction in endemic countries [78]. After decades of research the first dengue vaccine is now licensed, but, because the vaccine’s performance depends on serostatus, massive programmatic role out will be difficult [79,80]. The long-term safety follow up in the Phase 3 trials showed that the vaccine was efficacious and safe in those individuals who were seropositive at the time of vaccination, eg who had a previous dengue infection, but the vaccine increased the risk of severe dengue in those who were seronegative. On the basis of these new long-term safety data of the Phase 3 trials, WHO now recommends pre-vaccination screening for serostatus [79].

Complementing the development of methods and tools for dengue control, efforts were undertaken to disseminate products and findings. We conducted collaborative workshops with other dengue projects and organised seminars in different partner institutions. Many Masters and PhD students were trained as part of capacity building in research conduct and methodology, especially from low- and middle-income partner countries. A grand-finale dissemination event on dengue and dengue haemorrhagic fever was organised in conjunction with the Ministry of Health of Sri Lanka in Colombo, in February 2016, attended by local, regional and international health professionals. Further information is available at www.denguetools.com.

Detailed findings from DengueTools are presented in 47 peer-reviewed scientific publications, as listed in a Supplementary File. Moreover, Box 1 summarises DengueTools’ key deliverables.

**Box 1.** DengueTools’ key deliverables.

Research area 1: Laboratory-enhanced sentinel surveillance set up in Sri Lanka Prototype reverse transcription-recombinase polymerase amplification (RPA) developed Evaluation of RPA Research area 2: Cluster-randomised community-based trial completed with 1,800 school children Laboratory efficacy testing of permethrin impregnated clothing completed Qualitative studies and mathematical modelling studies done to support Economic value of potential preventive strategies assessed Research area 3: Sentinel surveillance for dengue in travellers returning to Europe was set up Mathematical models were developed for importation risk Mathematical models were developed for vectorial capacity for *Aedes aegypti* Predictive risk maps developed for Europe for both dengue and Zika virus Population growth determined as main driver for dengue resurgence Efficacy of Ultra-low volume insecticidal aerosols and thermal aerosols of deltamethrin for control of *Aedes albopictus* assessed in Southern France

Despite of global efforts the global scientific community still fails to stem the tide of dengue worldwide as dengue relentlessly continues to increase in incidence. Many research gaps still exist that the DengueTools consortium was not able to address. More implementation research, improved vaccines and vector control measures are needed to turn the tide.

## Outlook

Towards the end of DengueTools project funding, Zika virus (ZIKV) emerged as a new public health threat [81]. Given ZIKV`s relatedness to dengue viruses and its common vector, the European Commission asked us to extend our research to include ZIKV in the remaining months of our funded project time. Due to our expertise related to mathematical modelling, and vectorial capacity – developed through DengueTools – we decided to focus our research in this area. As ZIKV was rapidly spread via travellers [82–87], we modelled the risk of ZIKV importations from Brazil into Europe [69,88]. We analysed and overlaid the monthly flows of airline travellers arriving into European cities from Zika affected areas across the Americas, and the predicted monthly estimates of the basic reproduction number of Zika virus in areas where *Aedes* mosquito populations reside in Europe. Based on these analyses, we highlighted specific geographic areas and timing of risk for Zika virus introduction and possible spread within Europe to inform the efficient use of human disease surveillance, vector surveillance and control, and public education resources [89].

Furthermore, we modelled the timing and origin of ZIKV introduction into Brazil. Based on the force of infection in French Polynesia (where ZIKV caused an outbreak in 2014), ZIKV basic reproduction number in selected Brazilian cities, and estimated travel volume, we concluded that ZIKV was most likely introduced and established in Brazil by infected travellers arriving from French Polynesia in the period between October 2013 and March 2014 [90].

In 2016, the World Health Organization sought advice on whether ZIKV would be a risk at the upcoming Olympics in Brazil. We modelled the risk as not substantial [68,91,92] and advocated that the Olympics should not be cancelled. Subsequently, the virtual absence of ZIKV cases during the Olympics confirmed our predictive modelling.

When in early 2016, the European Commission launched a call for Zika research under Horizon 2020 Research and Innovation Programme, many of the DengueTools members together with new partners from Latin America assembled yet again to respond to the call under the consortium name ‘ZikaPLAN’. ZikaPLAN (grant agreement n° 734,584) was awarded €11.6 M in October 2016, as described elsewhere [93].

## Paper context

Dengue fever represents a numerically and geographically increasing global burden of disease, but often receives little attention. This review synthesises findings from DengueTools, a major international research programme funded by the European Commission, which was designed to develop strategies for dengue surveillance, diagnosis, prevention and forecasting.
