# Supplementary material for: Novel tools for the surveillance and control of dengue: findings by the DengueTools research consortium
Source: Glob Health Action. 2018 Dec 3;11(1):1549930. doi: 10.1080/16549716.2018.1549930 (PMC6282436; doi:10.1080/16549716.2018.1549930)
Supplement: Supplemental Material [file ZGHA_A_1549930_SM6127.pdf]

DengueTools Publications

Cross-cutting

Wilder-Smith A, Renhorn K, Tissera H, Abu Bakar S, Alphey L, Kittayapong P, et al. DengueTools: innovative tools and strategies for the surveillance and control of dengue. Global Health Action 2012;5:17273

Jaenisch T, Sakuntabhai A, Wilder-Smith A. Dengue research funded by the European Commission. Scientific strategies of three European dengue research consortia. PLOS Neglected Tropical Diseases 2013;7:e2320

Wilder-Smith A. Dengue vaccines: dawning at last? Lancet 2014;384:1327-9

Horstick O, Tozan Y, Wilder-Smith A. Reviewing dengue: still a neglected tropical disease? PLoS Negl Trop Dis. 2015 Apr 30;9:e0003632.

Tozan Y. Current issues in the economics of vaccination against dengue. Expert Rev Vaccines. 2016;15:519-28

Wilder-Smith A, Byass P. The elusive global burden of dengue. Lancet Infect Dis. 2016;16:629-631

Research area 1

Teoh BT, Sam SS, Tan KK, Johari J, Danlami MB, Hooi PS, Md-Esa R, Abubakar S. Detection of dengue viruses using reverse transcription-loop-mediated isothermal amplification. BMC Infect Dis 2013;13:387.

Harris CL, Sanchez-Vargas IJ, Olson KE, Alphey L, Fu G. Polymerase chain displacement reaction. BioTechniques 2013;55:93-97.

Teoh BT, Sam SS, Tan KK, Bashar Danlami M, Shu MH, Johari J, Hooi PS, Brooks D, Piepenburg O, Nentwich O, Wilder-Smith A, Franco L, Tenorio A, AbuBakara S. Early detection of dengue virus by use of reverse transcription- recombinase polymerase amplification. J Clin Microbiol 2015;53:830–837.

Thalagala N, Tissera H, Palihawadana P, Amarasinghe A, Ambagahawita A, Wilder-Smith A, Shepard DS, Tozan Y. Costs of Dengue Control Activities and Hospitalizations in the Public Health Sector during an Epidemic Year in Urban Sri Lanka. PLoS Negl Trop Dis. 2016;10:e0004466.

Tissera H, Amarasinghe A, Gunasena S, DeSilva AD, Yee LW, Sessions O, Muthukuda C, Palihawadana P, Lohr W, Byass P, Gubler DJ, Wilder-Smith. Laboratory-Enhanced Dengue Sentinel Surveillance in Colombo District, Sri Lanka: 2012-2014. PLoS Negl Trop Dis. 2016;10:e0004477

Louis VR, Montenegro Quiñonez CA, Kusumawathie P, Palihawadana P, Janaki S, Tozan Y, Wijemuni R, Wilder-Smith A, Tissera HA. Characteristics of and factors associated with dengue vector breeding sites in the City of Colombo, Sri Lanka. Pathog Glob Health. 2016;110:79-86

Tissera H, Pannila-Hetti N, Samaraweera P, Weeraman J, Palihawadana P, Amarasinghe A. Sustainable dengue prevention and control through a comprehensive integrated approach: the Sri Lankan perspective. WHO South-East Asia Journal of Public Health 2016: 5;106-12

Liyanage P, Tissera H, Sewe M, Quam M, Amarasinghe A, Palihawadana P, Wilder-Smith A, Louis VR, Tozan Y, Rocklöv J. A Spatial Hierarchical Analysis of the Temporal Influences of the El Niño-Southern Oscillation and Weather on Dengue in Kalutara District, Sri Lanka.. Int J Environ Res Public Health. 2016;13:E1087.

Tan KK, Azizan NS, Yaacob CN, Che Mat Seri NAA, Samsudin NI, Teoh BT, Sam SS, AbuBakar S. Operational utility of the reverse-transcription recombinase polymerase amplification for detection of dengue virus. BMC Infect Dis. 2018;18:169

Teoh BT, Sam SS, Tan KK, Johari J, Abd-Jamil J, Hooi PS, AbuBakar S. The Use of NS1 Rapid Diagnostic Test and qRT-PCR to Complement IgM ELISA for Improved Dengue Diagnosis from Single Specimen. Sci Rep. 2016;6:27663

Research area 2

Wilder-Smith A, Byass P, Olanratmanee P, Maskhao P, Sringernyuang L, Logan J G, et al. The impact of insecticide-treated school uniforms on dengue infections in school-aged children: study protocol for a randomised controlled trial in Thailand. Trials 2012;13:212

Massad E, Amaku M, Antoio F, Coutinho B, Kittayapong P, Wilder-Smith A. Theoretical impact of insecticide-impregnated school uniforms on dengue incidence in Thai children. Global Health Action 2013;6:20473

Murray N, Jansarikij S, Olanratmanee P, Maskhao P, Souares A, Wilder-Smith A, Kittayapong P, Louis VR. Acceptability of Impregnated School Uniforms for Dengue Control in Thailand: A Mixed Methods Approach. Global Health Action 2014;7:24887

Tozan Y, Ratanawong P, Louis VR, Kittayapong P, Wilder-Smith A. Use of Insecticide-Treated School Uniforms for Prevention of Dengue in Schoolchildren: A Cost-Effectiveness Analysis. PLoS ONE 2014;9: e108017

Banks S, Murray N, Wilder-Smith A, Logan J. Insecticide-treated clothes for the control of vector-borne diseases: a review on effectiveness and safety. Medical and Veterinary Entomology 2014; 28 (Suppl. 1):14–25

DeRaedt Banks S, Orsborne J, Gezan SA, Kaur H, Wilder-Smith A, Lindsey SW, Logan JG. Permethrin-Treated Clothing as Protection against the Dengue Vector, Aedes aegypti: Extent and Duration of Protection. PLoS Negl Trop Dis. 2015;9:e0004109

Kittayapong P, Olarantmanee P, Maskao P, Byass P, Lohr W, Gubler D, Wilder-Smith A. A school-based intervention trial using insecticide-treated school uniforms to reduce dengue infections in school-aged children. Tropical medicine & international health 2015;20 (Suppl. 1):114.

Ratanawong P, Kittayapong P, Olanratmanee P, Wilder-Smith A, Byass P, Tozan Y, Dambach P, Quiñonez CA, Louis VR. Spatial Variations in Dengue Transmission in Schools in Thailand. PLoS One 2016;11:e0161895

Orsborne J, DeRaedt Banks S, Hendy A, Gezan SA, Kaur H, Wilder-Smith A, Lindsay SW, Logan JG. Personal Protection of Permethrin-Treated Clothing against Aedes aegypti, the Vector of Dengue and Zika Virus, in the Laboratory. PLoS One 2016;11:e0152805

Kittayapong P, Olanratmanee P, Maskhao P, Byass P Logan J, Tozan Y, Louis V, Gubler D, JWilder-Smith A. Mitigating Diseases Transmitted by Aedes Mosquitoes: A Cluster-Randomised Trial of Permethrin-Impregnated School Uniforms. PLoS Neglected Tropical Diseases 2017;11:e0005197

Tozan Y, Ratanawong P, Sewe MO, Wilder-Smith A, Kittayapong P. Household costs of hospitalized dengue illness in semi-rural Thailand. PLoS Negl Trop Dis. 2017;11:e0005961

Research area 3

Massad E, Rocklöv J, Wilder-Smith A. Dengue infections in non-immune travellers to Thailand. Epidemiology and Infection 2013;14:412-417

Sessions OM, Khan K, Hou Y, Meltzer E, Quam M, Schwartz E, Gubler DJ, Wilder-Smith A. Exploring the origin and potential for spread of the 2013 dengue outbreak in Luanda, Angola. Global Health Action 2013;6:21822

Wilder-Smith A, Quam M, Sessions O, Rocklöv J, Liu-Helmersson J, Franco L, Khan K. The 2012 dengue outbreak in Madeira: exploring the origins. Eurosurveillance 2014;19:20718

Helmersson-Liu J, Stenlund H, Wilder-Smith A, Rocklöv J. Vectorial capacity of Aedes aegypti: Effects of temperature and implications for global dengue epidemic potential. PLOS One 2014;9:e89783

Rocklöv J, Lohr W, Hjertqvist M, Wilder-Smith A. Attack rates of dengue fever in Swedish travellers. Scand J Infect Dis 2014;46:412-7

Massad E, Wilder-Smith A, Ximenes R, Amaku M, Fernandez Lopez L, Bezerra Coutinho FA, Coelho GE, da Silva Jr JB, Struchiner CJ, Burattini MN. Risk of symptomatic dengue for foreign visitors to the 2014 FIFA World Cup in Brazil. Mem Inst Oswaldo Cruz 2014;109:394-7

Massad E, Burattini MN, Ximenes R, Amaku M, Wilder-Smith A. Dengue outlook for the World Cup in Brazil. Lancet Infectious Diseases 2014;14:552-3

Quam MB, Wilder-Smith A. Importation index of dengue to determine the most probable origin of importation. J Travel Med 2015;22:72

Quam M, Khan K, Sears J, Hu W. Estimating Air Travel-Associated Importations of Dengue Virus Into Italy. Journal of Travel Medicine 2015;22:186-193

Struchiner CJ, Rocklöv J, Wilder-Smith A, Massad E. Increasing Dengue Incidence in Singapore over the Past 40 Years: Population Growth, Climate and Mobility.PLoS One 2015;10:e0136286

Liu-Helmersson J, Quam M, Wilder-Smith A, Stenlund H, Ebi K, Massad E, Rocklöv J. Climate Change and Aedes Vectors: 21st Century Projections for Dengue Transmission in Europe, EBioMedicine 2016; 7:267-77

Ximenes R, Amaku M, Fernandez Lopez L, Bezerra Coutinho FA, Nascimento Burattini M, Greenhalgh D, Wilder-Smith A, Struchiner CJ, Massad E. The risk of dengue for non-immune foreign visitors to the 2016 summer Olympic games in Rio de Janeiro, Brazil. BMC Infect Dis. 2016; 16: 186

Massad E, Tan SH, Khan K, Wilder-Smith A. Estimated Zika virus importations to Europe by travellers from Brazil. Glob Health Action. 2016;9:31669

Rocklöv J, Quam M, Sudre B, German M, Kraemer MUG, Brady O, Bogoch II, Liu-Helmersson J, Wilder-Smith A, Semenza JC, Ong M, Kaasik Aaslav K, Khan K. Assessing Seasonal Risks for the Introduction and Mosquito-borne Spread of Zika Virus in Europe. EBioMedicine 2016;9:250-256

Amaku M, Azevedo F, Burattini MN, Coelho GE, Coutinho FA, Greenhalgh D, Lopez LF, Motitsuki RS, Wilder-Smith A, Massad E. Magnitude and frequency variations of vector-borne infection outbreaks using the Ross-Macdonald model: explaining and predicting outbreaks of dengue fever. Epidemiol Infect. 2016;144:3435-3450

Boubidi SC, Roiz D, Rossignol M, Chandre F, Benoit R, Raselli M, Tizon C, Cadiou B, Tounsi R, Lagneau C, Fontenille D, Reiter P. Efficacy of ULV and thermal aerosols of deltamethrin for control of Aedes albopictus in Nice, France. Parasit Vectors. 2016;9:597

Boubidi SC, Rossignol M, Chandre F, Tounsi R, Lagneau C, Fontenille D, Reiter P. Gender Bias in Insecticide Susceptibility of Aedes albopictus is Solely Attributable to Size. J Am Mosq Control Assoc. 2016;32:251-253

Louis VR, Phalkey R, Horstick O, Ratanawong P, Wilder-Smith A. Tozan Y and Dambach P. Modeling tools for dengue risk mapping - a systematic review. International Journal of Health Geographics 2014;13:50

Neumayr A, Muñoz J, Schunk M, Bottieau E, Cramer J, Calleri G, López-Vélez R, Angheben A, Zoller T, Visser L, Serre-Delcor N, Genton B, Castelli F, Van Esbroeck M, Matteelli A, Rochat L, Sulleiro E, Kurth F, Gobbi F, Norman F, Torta I, Clerinx J, Poluda D, Martinez M, Calvo-Cano A, Sanchez-Seco MP, Wilder-Smith A, Hatz C, Franco L; for TropNet22. Sentinel surveillance of imported dengue via travellers to Europe 2012 to 2014: TropNet data from the DengueTools Research Initiative. Euro Surveill. 2017;22:30433

Massad E, Amaku M, Coutinho FAB, Struchiner CJ, Burattini MN, Khan K, Liu-Helmersson J, Rocklöv J, Kraemer MUG, Wilder-Smith A. Estimating the probability of dengue virus introduction and secondary autochthonous cases in Europe. Sci Rep. 2018;8:4629
